# Innovative Biochar-Based Composite Fibres from Recycled Material

**DOI:** 10.3390/ma14185304

**Published:** 2021-09-14

**Authors:** Sandra Lepak-Kuc, Mateusz Kiciński, Przemyslaw P. Michalski, Krystian Pavlov, Mauro Giorcelli, Mattia Bartoli, Malgorzata Jakubowska

**Affiliations:** 1 Institute of Metrology and Biomedical Engineering, Faculty of Mechatronics, Warsaw University of Technology, Sw. Andrzeja Boboli 8, 02-525 Warsaw, Poland; mateusz.kicinski@gmail.com (M.K.); maljakub@mchtr.pw.edu.pl (M.J.); 2 Solid State Ionics Division, Faculty of Physics, Warsaw University of Technology, Koszykowa 75 Street, 00-662 Warsaw, Poland; przemyslaw.michalski@pw.edu.pl; 3 Centre for Advanced Materials and Technologies (CEZAMAT), Warsaw University of Technology, 02-822 Warsaw, Poland; K.Pavlov@cezamat.eu; 4 Department of Applied Science and Technology, Politecnico di Torino, Corso Duca degli Abruzzi, 10129 Torin, Italy; mauro.giorcelli@polito.it; 5 Consorzio Interuniversitario Nazionale per la Scienza e Tecnologia dei Materiali (INSTM), Via G. Giusti 9, 50121 Florence, Italy; mattia.bartoli@polito.it; 6 Fondazione Istituto Italiano di Tecnologia, Via Livorno 60, 10129 Turin, Italy

**Keywords:** polymer–matrix composites, electrical properties, mechanical properties, physical methods of analysis

## Abstract

Carbon materials are becoming crucial in several industrial sectors. The drawbacks of these materials include their high cost and oil-based essence. In recent years, recycled materials have become possible alternative sources of carbon with several advantages. Firstly, the production of this alternative source of carbon may help to reduce biomass disposal, and secondly, it contributes to CO_2_ sequestration. The use of carbon derived from recycled materials by a pyrolysis treatment is called biochar. Here, we present composite materials based on different biochar filler contents dispersed in several thermoplastic polymer matrixes. Electrical conductivity and tensile break strength were investigated together with the material characterisation by DTA/TGA, XRD, and scanning electron microscopy (SEM) imaging. Materials with good flexibility and electrical conductivity were obtained. The local ordering in composites resembles both biochar and polymer ordering. The similarity between biochar and carbon nanotubes’ (CNTs) XRD patterns may be observed. As biochar is highly cost-effective, the proposed composites could become a valid substitute for CNT composites in various applications.

## 1. Introduction

Rapidly increasing developments in industry and science over the last few decades have resulted in high demand for new materials for various applications. The high price of the limited number of commonly used materials and the miniaturization of new technological devices have played a significant role in the direction of the research for new materials [1,2]. These needs have opened up a broad path of development and intensified research for carbon nanomaterials. The best-known nanocarbons include graphene, carbon nanotubes, carbon black, and fullerenes, useful for their unique characteristics and very often considered the solution to many problems [3,4,5,6,7]. As well as these carbon nanomaterials, a considerable number of new ones are constantly being discovered or synthesized. The following varieties of nanocarbon are also frequently used: (i) carbon nanodots, offering various applications such as electronics and optoelectronics, which should be noted [8]; (ii) mesoporous carbon nanomaterials, with applications in biomedical engineering such as drug delivery [9] or chemical adsorbents [10]; and (iii) the group of lignin-based, starch-based, and bark-based carbon nanomaterials, classified as so-called graphene nanoplatelets, with proven high adsorption properties [11].

One of the core usages of carbon nanomaterials is in polymer composites. There are many different kinds of composites with a variety of applications, but above all, with different components. The most commonly researched composites of carbon nanomaterials are the ones based on graphene and carbon nanotubes. Such composites find application in many areas such as medicine, Li-ion batteries, and especially electronics [12,13,14]. Though nanocarbon composites can offer many useful properties, there are a couple of issues that have to be highlighted. Despite the great electrical properties of carbon nanomaterials themselves, obtaining good electrical conductivity while maintaining reasonable flexibility in the case of composites is often problematic. Furthermore, economic affordability is usually required for composite materials. Here, another problem arises, namely the high price of basic carbon nanomaterials.

Biochar represents an interesting alternative to combine affordability and performance improvements [15]. Biochar is the solid residue recovered from the thermal conversion of biomasses at high temperature in an oxygen-free atmosphere [16].

In recent years, biochar-based composites and their derivatives have been studied, leading to the synthesis of interesting and functional materials. It was shown that biochar can be found as an equivalent to carbon nanotubes in many solutions [17]. Composites containing 20 wt.% of biochar are reported to have similar electrical properties to those containing 4 wt.% carbon nanotubes [17]. The addition of biochar to composites can improve not only electrical properties, but also mechanical properties. Epoxy composites containing biochar, even in such small amounts as 2 wt.%, showed much higher ultimate tensile strength, compared not only with epoxy itself but also with composites of epoxy with carbon nanotubes [17,18]. Biochar is also characterized by the possibility of denser packing in the composite than other carbon nanomaterials [17,18,19].

For several years, conductive materials made of carbon have been considered essential for the development of electronics and related fields. The main emphasis is on highly conductive pure-nanotube fibres [20,21,22]. However, the development of areas with lower electrical performance requirements, like smart-textiles, implies a need for economical, electrically conductive composite fibres [23]. In line with these trends, the present work focuses on the production of flexible and electrically conductive biochar-polymer fibres. We present thorough research on the production of a functional, electrically conductive composite based on biochar.

## 2. Materials and Methods

### 2.1. Materials

Derived from recycled cotton fibres, Biochar is synthesized as described [24,25]. To produce the composite in the form of fibres, the screw extruder (purpose-designed and assembled at the Faculty of Mechatronics, WUT) was used. This technology requires limiting selected polymers to thermoplastic materials. The choice of which polymers to test took into account their mechanical and electrical properties. Composites of biochar with the following polymers were made: thermoplastic polyurethane (TPU), acrylonitrile butadiene styrene (ABS), polylactide (PLA), ethylene/vinyl acetate (EVA), and poly (methyl methacrylate) (PMMA). All polymers chosen were thermoplastic because a screw extruder was used in the fibre shape-forming technique. PMMA (average molar mass ~350,000 g/mol) was purchased from Sigma-Aldrich (Darmstadt, Germany). TPU (shore hardness of 35 A) was purchased from BASF (Lemförde, Germany). EVA (shore hardness of 80 A) was purchased from Repsol (Madrit, Spain). PLA (shore hardness of 80 D) and ABS (Rockwell R-scale hardness of 110) were purchased from Finnotech (Katowice, Poland).

#### Composite Preparation

The process of composite fibres’ manufacturing was carried out step by step as follows. For every polymer, an appropriate solvent was chosen. The weighed (125A Precisa laboratory balance) biochar was sonicated in the solvent for half an hour on a VCX130FSJ Sonics and Materials INC device (Sonics, Newtown, USA). The sonication process was held in the presence of AKM-0531 surfactant from NOF Corporation, Tokyo, Japan in an amount of 3 wt.% relative to the functional phase. The polymer was then added to a mixture and sonication was continued for ten more minutes. Next, the mixture was mixed overnight on a magnetic stirrer Heidolph Mr Hei-Standard (Heidolph Instruments, Schwabach, Germany). The obtained suspension was dried in an FCF 7-SM 2002 Czylok oven (Czylok, Jastrzebie-Zdroj, Poland) until complete evaporation of the solvent. This procedure was proposed previously [23] and allowed for the proper distribution of the functional phase in the composite. The cooled composite material was cut into smaller pellets, suitable for the extrusion process.

### 2.2. Methods

Electrical characterization.

To determine the samples’ electrical conductivity, fibres were cut into 10 cm long pieces and their length, diameter, and weight were measured. Then, silver paste was applied onto their ends and, after drying, the resistance was measured. All of these measurements were performed for five different samples and the average was computed for each tested composite material.

Mechanical characterization.

Samples of 4 cm length were placed in the QC-506 M2 Cometech ripper (Cometech, Taichung City, Taiwan) for breaking strength testing in such a way that the stretched fragment was 2 cm long. The samples were then loaded with increasing mechanical force starting at 0.3 N.

Thermal analysis (DTA/TGA).

Samples of all tested materials were crushed into fine-grained dust and differential thermal analysis (DTA) and thermogravimetric analysis (TGA) were performed. DTA/TGA were conducted in an argon atmosphere to avoid sudden reactions and processes, like carbon oxidation. The measurements were conducted on TA Instruments Q600 thermogravimeter (TA Instruments- Waters LLC, New Castle, DE, USA) and analysed in TA Universal Analysis. A container with a capacity of 40 microliters was loaded with composite dust and measurement was conducted with a heating rate of 10 K/min, under an argon flow of 100 mL/min, from room temperature up to 600 °C.

X-ray diffractometry.

As in the DTA/TGA measurements, samples of all tested materials were crushed into fine-grained dust, and then X-ray diffraction (XRD) was performed on an Empyrean Panalytical diffractometer (Malvern, GB). The measurement was carried out at room temperature. HighScore Plus software (version 4.7, 2017, PANalytical B.V., Almelo, Netherlands) from Malvern Panalytical was used to identify and match the peaks. Crystallographic database PDF4 was used while analysing XRD results.

Morphological characterization.

Scanning electron microscopy was performed on a high-resolution Carl Zeiss Auriga 60 scanning electron microscope (Jena, Germany).

## 3. Results and Discussion

### 3.1. SEM Analysis

**Figure 1:**
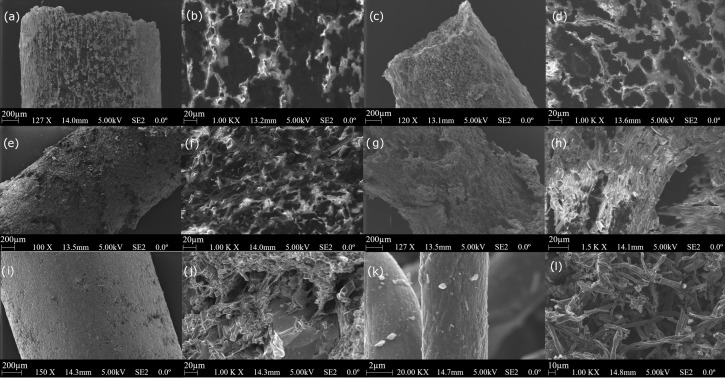
Scanning electron microscope images of (**a**,**b**) PMMA-biochar, (**c**,**d**) PLA-biochar, (**e**,**f**) ABS-biochar, (**g**,**h**) EVA-biochar (**i**,**j**), TPU-biochar (BC 60%) composites, and (**k**,**l**) biochar-derived from recycled cotton fibres.

Scanning electron microscope images (Figure 1) were performed on the surface of the fibres as well as on the cross sections of the composite samples to examine the structure of fabricated fibres and define the distribution of biochar in the composite structure.

The presented SEM images of the fibres’ cross sections show high isotropy of the composite structures. However, different roughness, both externally and internally, can be noticed for different composites. Composites containing PMMA and TPU polymers had a smoother surface than those made of EVA, PLA, or ABS. The cross-section images indicate the relatively even distribution of biochar material in the polymer matrix. The images of biochar powder (Figure 1k,l) show the structure of the material in the form of micro and nanowires. The material’s tendency to agglomerate into larger clusters is also visible. This allows us to affirm the correctness of the composites’ production procedure starting with the sonication of the functional phase in the presence of a surfactant.

### 3.2. Electrical Results

One of the major goals of the presented work was that of obtaining an electrical conducting composite. Thus, efforts were made to discover what content of biochar was needed in the extruded fibre composite form to ensure electrical conductivity. For this purpose, a series of samples with a functional phase content in the range of 10–90 wt.% was made. For every sample, the percentage content was increased by 10% compared with the previous one. TPU was chosen as polymer, because of our previous work on CNT composite fibres [23]. For the samples containing 10, 20, and 30 wt.% of biochar, the percolation threshold was not exceeded. On the other hand, the content of 70–90 wt.% composites showed excessive brittleness, already with manual processing. Considering the differentiation of the tested polymers in terms of their elasticity, the functional phase content of 50 wt.% was chosen as the optimal value for further comparison of the composites. For every polymer, a composite fibre with 50 wt.% of biochar was produced.

Two types of conductivity were calculated to thoroughly investigate the effect of a given polymer density and the packing density of the functional phase. Those were absolute electrical conductivity and specific electrical conductivity. Absolute electrical conductivity was determined with the following formula:

Σ = L/(R × S),(1)

where L is the sample length, S is a sample cross-sectional area, and R is sample electrical resistance. Specific electrical conductivity was determined with the following formula:

σ′ = L^2^/(R × m), (2)

where L is the sample length, m is sample mass, and R is sample electrical resistance.

The average absolute conductivity and specific conductivity values were calculated for each of the materials and are presented in Table 1.

The results presented in Table 1 show that the highest electrical conductivity, both absolute and specific, was obtained for 60 wt.% of biochar. This is consistent with the predictions that, the higher the content of the functional phase, the better the electrical parameters of the composite. Comparison of different composites with the same filler content showed that the highest electrical conductivity, both absolute and specific, was exhibited by the composite based on PMMA. The lowest values of both electrical conductivity values were measured for a PLA-based composite. These values were even lower than a 40 wt.% TPU-based composite.

### 3.3. Mechanical Results

**Figure 2:**
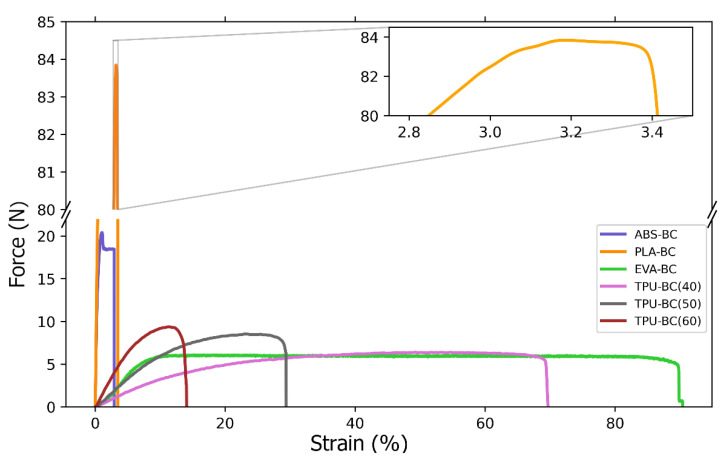
Stress and strain curves of composite fibres containing various polymers and biochar (BC) as a functional phase. Composites with ABS, PLA, and EVA contained 50% by weight of biochar, while for the TPU-biochar composite, the results for the three percentages of functional phase content are shown: 40%, 50%, and 60%.

While designing electrically conductive composite fibre, attention must also be paid to the mechanical properties. As shown by preliminary tests, the content of more than 70 wt.% of the functional phase is associated with exceeding the minimum strength of the composite required for its processing, and thus the functionality as well. Composite fibres were tested for breaking strength. Both the comparison of different polymer bases with the same biochar content of 50 wt.% and the different percentage packing of the functional phase with the TPU as a polymer were examined (Figure 2). The PMMA composite exhibited excessive brittleness for the proposed measurement settings and did not withstand the initial load of 0.3 N.

After stretching samples to the breaking point, the maximal elongation and maximal loading force were calculated from the data obtained from mechanical treatment and the sizes of samples measured before stretching (Table 2).

As the results of the mechanical measurements (Table 2) show, the content of the functional phase in the form of a powder limits the flexibility of the composite. Comparing the three different contents of the functional phase in the TPU matrix, a clear decrease in elongation with an increase in the powder phase content can be seen. It should also be noted that, as the biochar content increases, the maximum stress that the samples can withstand also increases from about 3 MPa to about 5 MPa.

The influence of the polymer properties on the flexibility and strength of the composite was also clearly visible. EVA and TPU used in this work can be easily described as “rubber-like” owing to their softness and flexibility. For these materials, the highest elongation of composite specimens was obtained. On the other hand, ABS, PLA, and PMMA are characterized by high hardness, which improves the mechanical strength of the composite, but reduces its flexibility. This was especially noticeable in the case of PMMA, where the composite did not withstand even the minimum required stress set by the testing machine.

This behaviour was also observed by Bartoli et al. [24] and it was understandable owing to the creation of a micrometric network of the fibres, leading to an increase in brittleness.

It is also worth comparing the results obtained for the TPU matrix composite with previous research on composite fibres based on carbon nanotubes [23]. For the composite nanotube fibre with the same diameter as the fibres presented in this work, the force of 18 N was obtained, which, in terms of tensile strength, gives a result of about 10 MPa, two times higher than for the biochar material. The maximum elongation shows an inverse relationship. With the same weight packing, 40% of the functional phase, the CNT-TPU fibres showed an elongation of 35%, while for the biochar-TPU fibres, more than 70% was achieved. In a rough comparison with fibres loaded with 20 wt.% CNTs, biochar containing composites displayed far lower conductivity, and this was understandable because of the significant difference in the conductivities of the fillers themselves. In line with Noori et al. [25], biochar produced at 1000 °C showed an electric behaviour close to that of metal, but it was still far from the conductive values of CNTs. Another advantage of the Biochar material is seen here, namely the possibility of its greater compaction in the composite. One could thus point to the interchangeability of the fibres based on these carbon materials concerning various potential applications.

### 3.4. Thermal and Thermogravimetrical Results

**Figure 3:**
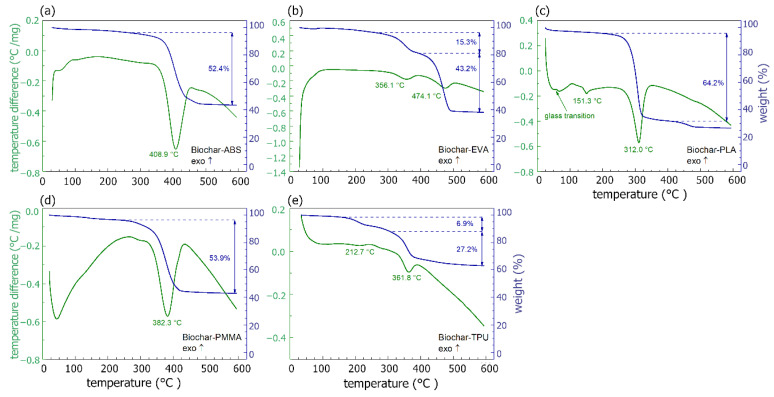
DTA (green lines) and TGA (blue lines) measured for biochar composites: (**a**) ABS-biochar, (**b**) EVA-biochar, (**c**) PLA-biochar, (**d**) PMMA-biochar, and (**e**) TPU-biochar (60 wt.% biochar).

TGA and DTA measurements are known tools for studying a material’s thermal stability. Both DTA and TGA were conducted on electrically conductive samples (containing PMMA, PLA, ABS, EVA, and TPU). From the composites based on TPU, the one with 60 wt.% of biochar was tested, as it exhibited the best electrical conductivity values while providing an acceptable mechanical strength at the same time. The characteristics obtained allowed for the determination of characteristic temperatures and mass loss (Figure 3).

In the range of low temperatures, below 100 °C, changes in DTA signals were observed, related to a slight decrease (a few %) in mass, suggesting that evaporation of materials’ contaminations such as solvent residuals took place. In the case of PLA-biochar composites, the endothermic effects at ca. 60 °C and 151 °C may be attributed to polymer glass transition and melting, respectively [26]. For TPU-biochar, a broad peak around 213 °C, with the corresponding mass change of ca. 6.9%, is observed. It may be related to the melting process of hard, thermoplastic segments of TPU. In addition, the process of volatile evaporation starts at ca. 150 °C, which experimental data bore out [27].

In the temperature range of 300–480 °C, depending on the composite, a DTA endothermic peak corresponding to high mass loss in the TGA curve was observed. This was the effect of polymer evaporation from the composite, which was expected [28,29,30,31]. Differences occurred in the percentage mass loss of material during evaporation. Those differences were caused by the materials’ manufacturing process. The double sonification, mixing, and magnetic stirring resulted in the more homogeneous composition of biochar in the polymer matrix, lowering samples’ resistivity. However, such a multi-stage process may cause some losses, for example, through the settling of the materials on vessel walls. Good thermal stability (up to 100 °C) of tested materials was also observed.

### 3.5. XRD Measurement Results

**Figure 4:**
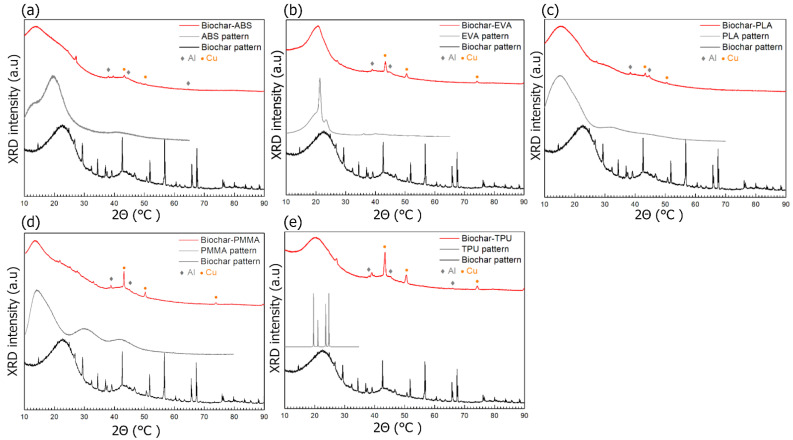
XRD results of (**a**) ABS-biochar, (**b**) EVA-biochar, (**c**) PLA-biochar, (**d**) PMMA-biochar (50 wt.% biochar), and (**e**) TPU-biochar (60 wt.% biochar). Experimental diffractograms are marked with the red line. The grey line depicts the polymer pattern taken from the ICDD database, while the black line being an experimental pattern of biochar.

XRD measurements were carried out in order to discern the presence of any contaminations and to determine the phase composition. XRD measurements were conducted on the following composites: ABS-biochar, EVA-biochar, PLA-biochar, PMMA-biochar, and TPU-biochar (60 wt.% of biochar). The results are presented in Figure 4.

The diffractograms of composites (red lines) presented in Figure 4 show the continuous and broad-peak shape change of signals. This indicates a lack of crystallinity of composites. For comparison, the experimental pattern of pure biochar (black lines) and polymer patterns (grey lines) are presented. Polymer patterns were taken from the ICDD PDF-4 crystallographic database. The PDF card numbers are as follows: 00-060-1508 (ABS), 00-062-1287 (EVA), 00-064-1622 (PLA), 00-064-1603 (PMMA), and 00-058-1367 (TPU). In the case of the first four polymers, raw experimental diffraction data were available in the database and were used for presentation. In the case of TPU, merely the peak positions for crystalline polymer were indexed, so only those were presented for comparison. The resemblance between broad-peak shapes in composites and biochar/polymer patterns suggests the structural similarities between them. The local structure of composites has features observed in biochar and polymer patterns alone.

In all diffractograms, low-intensity peaks may be observed, indicating the presence of crystalline impurities. Copper contamination for all composites and additional aluminium contamination for all composites, except ABS-biochar, was noticeable. This was likely the result of the fibres’ crushing process. To conduct the analysis, the dust form of materials was necessary. As tested materials were mostly highly elastic, obtaining the dust form was a problematic process, which required the use of various abrasive tools made of metals. The peaks of metals visible in the presented diffractograms can be easily linked to the tools used. Therefore, with great certainty, the impurities were introduced at this stage, thus it can be argued that the fibre form, which has not undergone the process of crushing, is free from such impurities. Except for metallic phases, other low-intensity peaks are present in composites’ patterns. They could not be ascribed to any crystalline phase and to the biochar phase.

It is also worth noticing that the biochar experimental pattern resembles the pattern of CNTs. The typical diffraction pattern of CNTs consists of two broad peaks centred at 2θ ≈ 24° and 43° [32]. Similar structures are observed in the biochar pattern, which may suggest a resemblance between biochar and CNTs’ atomic and molecular structures.

## 4. Conclusions

We have reported homogeneous, biochar-based composites in a fibre structure. Composites in ABS, EVA, PLA, PMMA, and TPU polymer matrixes were tested for electrical, mechanical, structural, and thermal properties. The different contents of the functional phase were also taken into consideration, paying particular attention to obtaining the highest possible electrical conductivity while ensuring practicable elasticity for potential applications.

Examination showed that there was a strict correlation between biochar content in the composite and its electrical conductivity, which rose in line with higher biochar content. There was also a link between the electrical and mechanical parameters, indicating poorer electrical properties with a higher elasticity of the fibres. This was a predictable result owing to the powdery form of the functional phase, which meant that, the higher its content in the composite, the more brittle it was. It was found that the best electrical conductivity reached the same level as the composite biochar-TPU (60 wt.%).

Thermal examination confirmed the thermal stability of the biochar/polymer composites. XRD measurements revealed similarities in local structure between composites and their substrates and between biochar and CNTs. The analysis of the results showed only a slight amount of impurities in the material, which did not cause significant uncertainty in their composition.

The fibres were characterized by an even distribution of biochar material in the polymer matrix, which confirmed the correctness of the procedure employed for preparing composites, based on sonication of the functional phase in the presence of a surfactant.

Our results indicate that, in some applications, biochar can be considered a great alternative to the currently widely used carbon nanomaterials, such as carbon nanotubes or graphene. Furthermore, there are the twin advantages of the low cost of material and the low impact on the environment of the biochar synthesis process. The composite fibres presented will be useful for many applications, both in industry and new technologies. Carbon fibre-like materials are generally at the centre of attention in many fields. Their future is seen, for example, as a replacement for electric wires. The area of smart clothes is worth special attention in the context of the applications of the proposed biochar-based fibres. In this field, highly flexible wires are sought that additionally do not exhibit exorbitant electrical requirements [33]. Additionally, all the composites proposed in the study are based on thermoplastic polymers commonly used in 3D printing technology. In the last decade, there has been an intensified work on the production of carbon filaments [34,35,36]. They are believed to have exceptional potential for the production of three-dimensional electronics and for applications in printed electronics [37,38]. A particular advantage of biochar in this context is the possibility of its dense packing in composites, which we have indicated in this paper.

## Figures and Tables

**Table 1:** The average values of absolute and specific conductivity measured for various polymers in biochar composite fibre.

Polymer	Biochar Content (wt.%)	Average Absolute Conductivity (S/m)	Average Specific Conductivity (S m^2^/kg)
TPU	40	3.14 × 10^−2^	1.99 × 10^−5^
TPU	50	5.17 × 10^−1^	3.39 × 10^−4^
TPU	60	11.9	7.44 × 10^−3^
ABS	50	7.73 × 10^−2^	5.3 × 10^−5^
EVA	50	2.01 × 10^−1^	1.44 × 10^−4^
PLA	50	1.03 × 10^−2^	8.49 × 10^−6^
PMMA	50	1.70	1.36 × 10^−3^

**Table 2:** Results of mechanical measurements of the composite fibres.

Polymer	Biochar Content (wt.%)	Maximal Elongation of the Sample (%)	Ultimate Tensile Strength (MPa)
TPU	40	70%	3.28
TPU	50	29%	4.81
TPU	60	14%	5.10
ABS	50	3%	11.89
EVA	50	90%	3.11
PLA	50	3%	47.56

## Data Availability

All the data is available within the manuscript.
